# Covid-19 Outbreak – Immediate and long-term impacts on the dental profession

**DOI:** 10.12669/pjms.36.COVID19-S4.2698

**Published:** 2020-05

**Authors:** Fazal Ghani

**Keywords:** Covid-19 outbreak, Impact on dentistry, Dental professionals, Dentistry

## Abstract

The health professions and systems have been challenged evoking heightened reactions around the globe as response to Covid-19. While most heavily impacted, the role of the dental professionals in preventing the transmission and responding to its long-term impacts on dentistry is critically important. This report, while outlining the immediate impact that the Covid-19 outbreak currently has on dental healthcare professionals, it also looks at some heavier impacts that this outbreak might have on the profession of dentistry. As such this manuscript offers some suggestions and recommendations based on personal feeling.

## INTRODUCTION

Since the outbreak of Covid-19 and its declaration by the WHO as a public health emergency of international concern (PHEIC), an exponential interest to know more about, the transmission, pathogenicity, prevention, treatment, the novel coronavirus-2 or SARS-CoV-2 and the resulting pandemic is evident.^1^-^7^ Guidelines, of the international and local medical agencies, especially those to control the airborne transmission risk of the novel-coronavirus (SARS-CoV-2) resulting in Covid-19, have been interpreted not only varyingly in different countries, but ironically they had been silent in guiding dentistry.^6^ This silence did continue, till the publications of dental academics in Wuhan, China.^1^-^2^ No doubt, the response of the dental professionals and its impact on them is very much visible.^1^-^2^, ^5^-^7^ This report, while outlining the immediate impact that the Covid-19 outbreak currently has on dental healthcare professionals, it also looks at some heavier impacts that this outbreak might have on the profession of dentistry. As such this manuscript offers some suggestions and recommendations based on personal feeling.

### Impact on finances and employment

A most recent survey involving some 20 thousand US dentists is reflecting more despair and little hope.^7^ In fact, the reporting of the vast majority of dental practices in USA of a lower volume of both patient’s turns and financial collections is certainly alarming. To explain it, approximately nine out of ten dental practices had less than a quarter of their typical patient volume with 82% dental practices having had less than a quarter of typical income and revenue.^7^ The decrease in earning and patients flow could certainly affect the ability of most practices to pay their employees. In fact, the inability of paying the employees over a longer time can be further sensed from the finding that 28% of dentists remained unable to pay their staff during the 3^rd^ week of March 2020 with another 45% who managed partial payment. Just about 27% provided full pay to their employees.^7^ The US situation given here applies to everywhere in the world, where almost all dental practice have been shut either voluntarily or by governmental orders of sheltering at homes.

Whether to practice or not, the findings, were varying. In most US states, dental practices despite having taken the precautions seriously, only as little as 1.2% did business as usual.^7^ Furthermore, the patients’ flow was drastically affected with few patients reporting only for what can be described as urgent dental care. The American Dental Association (ADA) intended to continue the survey on a biweekly basis, to keep monitoring the effects of the pandemic. Similarly, most of the professionals (>11,000) agreed to respond.^7^ However, despite this, there is some hope among the dentists amid most dentists currently dealing with stresses and uncertainties especially about the dentistry as a viable and vibrant profession. In light of these observations and the tremendous concerns of the Covid-19 impact on the profession of dentistry,^1^-^7^ I personally feel the need to elaborate upon what will come next and how to respond?

### Impact and concerns related to dental practice and dentistry

There is apparent likelihood that dentists and other members of the dental team are having an increased risk for catching / and or transmitting this life threatening viral infection and the other involving the respiratory system because of their involvement of seeing high volume of patients requiring close contact with them.^2^-^3^,^5^-^6^ This virus can cause COVID-19 by remaining airborne through aerosols formed during medical procedures or indirectly through saliva.^8^,^9^ It is also capable of causing infection with transmission through contact with an asymptomatic patient.^10^ This has created a lot of fear and awareness among many, about dentistry. As such, I read and witness the uncertainty and also I hear from existing dental students as well as the many would be dental students and their parents, to think of or pursue dentistry as career. Hence, it is anticipated that even in the coming year or so, this reluctance will continue toward seeking admission in dental institutions. This shall, obviously, have a severe adverse effect, on those currently thinking to invest in the dental education and dental industry sectors. The implication for dental education and training are also considerable. In fact, it is likely to see fewer people as patients to be visiting dental practices for elective treatments. Similarly, it is likely to see fewer dental students seeking dental education and training in the existing training institutions deficient in terms of airborne infection isolation infrastructure. While many investors are likely to be responding addressing these issues, for some it will remain beyond their resources. Dental education will soon be seen as very costly unaffordable by students and their parents. Additionally, it is likely that reduced demand for dental products and equipment will also impact on new investment in dental education and dental healthcare industry.

Despite the explosion of information available online and through social media, it is still difficult to identify reliable research evidence and guidance. As such most of the decisions made have simply moral ground with no evidence from research.^6^ In order to provide education and training to *“Tomorrows’ Dentists”*, there is an urgent need to revise dental curricula and training programs with increased emphasis on learning and practical training related to topics that could help enlarging understanding of the infectious diseases, tele-dentistry, designs of dental clinics and dental teaching hospitals that is based on evidence of relevance to safe dentistry and safe dental practice.

The rising boom for orthodontics / elective restorative and cosmetic dental services will no more continue and perhaps considered absolutely unnecessary by many people. Gone seem to be the “prophy mills’ where dentists / hygienists have been working in a ‘fast food’ dental environment without adequate time for the provision of safe care.^11^ A need is obvious to consider a new definition of what will make a dental patient. The irony is, we have made the mentioned elective cosmetic and restorative dental services so expensive for the ordinary and the less affluent that most of them have already become convinced about the very nature of these dental treatments as not only unnecessary but also as un-afforded luxurious therapies.

Perhaps, more worrying is that in the coming time, our slogans highlighting and emphasizing the link between oral health and systemic health will be ignored by many people as a reason for seeking dental treatment and maintaining oral and dental health. Furthermore, our preaching of the impact of teeth of the vibrant and pleasing look and smile, on the quality of life, better prospect for social engagements and employment will not convince our patients anymore to seek dental consultation and dental care services for achieving these outcomes considered due from dentistry. As such, it is likely that we may see less dentistry on the globe. Hence many existing dental professionals have already started thinking of leaving, with yet many more to be thinking of quitting their beloved dental profession permanently and opting for alternative jobs and career fields. There have been many reports but one from UK, suggest this when they considered deployment of its many dental healthcare professionals including: dentists and dental nurses in hospital practice; dental foundation trainees; core trainees; clinical academics for new roles with many more experiencing deployment to other new roles.^6^ Dental hospital buildings have been considered to undergo reconfiguration to host medical care with only specialists in oral and maxillofacial surgery kept engaged to provide urgent dental and facial trauma and oncology care.^6^ More recently, tens of thousands of dental practice owners in USA, have been projected to be looking into selling their businesses within the next year or two.^13^ Some have been reported to even have started the process by seeking appraisals, speaking with advisers, or placing their practices on the market.^13^

### Recommendations and suggestions

In such an uncertain and truly testing situation, the responsibility of senior dental professionals is to come forward and help and save dentistry as a profession. Dental professionals are required to feel encouraged and to remain innovative and imaginative in this unprecedented period. This, they can do by considering how to help and work and support dental industry in their efforts of quickly coming up with feasible new universal design full-fledge airborne infection isolation dental surgery clinic (AIIDSC). Though, we hold, in dentistry, to the principle of universal precautions for cross-infection control based on an understanding that we may not know whether a patient has or has not the potential for disease transmission, our working environment must have all the safety design features in the first place. The comment of the director of an intensive care unit (ICU) in Wuhan, China are certainly noteworthy, whereby he stated that if he could do it all over again, he would have pushed health care authorities harder in hospital intensive care units to better establish best practices for critical care.^11^ Surely, this does, also very much apply to dental clinics and dental hospitals. The US Occupational Safety and Health Administration (OSHA) has issued COVID-19 workplace recommendations.^12^ These recommendation especially focus on the need for employers to implement engineering, administrative and work practice controls, in addition to personal protective equipment (PPE).^12^ These also include an infectious disease preparedness and response plan and actions.^12^

Surgical facemasks are not effective to prevent submicron-sized particles and the microbes and viruses present in blood and saliva aerosolized produced in during dental procedures.^14^-^15^ While it is possible to have effective vaccination against TB, measles and Hepatitis B, this is, unfortunately, at yet not the case with viruses like Ebola, SARS, MERS-CoV and COVID-19.^16^ Most recently, a number of ‘weak-points’ in the dental practice with respect to viral transmission necessitates drastic measurement.^17^ To protect from the hazards of aerosol, generated in dentistry, personnel must use N95 and FFP3 facemasks, face shield, goggles and over-garments for prolonged periods. These will cause discomfort, vision impairing, and preventing dentist-patient communication. Field vision impairment with the PPEs on face, will become even more concerning, for dentists using prescription glasses and dental loupes. Demonstrating tooth-brushing technique to patients needs using Oral-B Test Drive Unit every day. This cannot be done while wearing the enhanced PPEs. A powerful spray mist suction system (300L/min) to suck the aerosol must be used for which the needed four-handed approach to dentistry is needed.^18^ A deeper look into the clinic design with efficient airflow, air-conditioning and ventilation system and adequate positioning of windows will also be needed.^17^ All these will add huge cost to the dental practice.

To keep continue the student flow in for training, we need to assure them that we have, for training them, a dental teaching hospital environment with safe design features. This will include features to control any infection but specifically those for controlling and prevention of airborne viral infections. Without new design dental clinics / surgeries and teaching dental hospitals, dental healthcare professionals, students and dental patients will be reluctant to come as users. Certainly the financial and other implications for doing so in designing dental teaching hospitals will be too high. Once we have come-up with new design infection-free and safe dental practices and teaching dental hospitals, that are fool-proof capable in preventing the transmission of airborne, blood-borne, contact-borne viral and bacterial infections, only then the users (people) of these facilities will start feeling convinced and confident in treating their dental ailments and seeking for dental facial enhancements, opting for taking dentistry as a profession and investors in dental education and industry. This kind of response, from the custodian of the dental profession is very much needed to restore trust and keep motivated all stakeholders (dental patients, current young dentist workforce, dentists, current trainees and students) to remain in the profession and the incumbent students to consider opting for dentistry as a career.

## CONCLUSIONS

1. A sorry and confused situation for the dentistry shall indeed continue till the development and availability of a point-of-care quick serological Covid-19 testing suitable for dental practice setting and a vaccine for Covid-19 having proven long-lasting efficacy. That is what all in the dental profession are desperately looking forward to.
2. In addition we must work hard to find better solutions to keep our much needed profession stay on thriving and to remain on the frontline of healthcare.
3. It is high time that we keep supporting each other in our professional family including our students, support and help patient with reduced contact, restrict the generation of aerosols and use the best personal protective equipment (PPE).
4. We must also look out for our own mental health and wellbeing, and that of each other.
5. We all know there should be strong belief to distance fear well away from the hope we currently have. In this regard, the good news of the coming back to normal of the routine dental care in Wuhan, China, since its suspension last January 2020, is certainly encouraging but the effects arising from this, if any, need to be strictly followed over a period.
